# Nomogram to predict risk for early ischemic stroke by non-invasive method

**DOI:** 10.1097/MD.0000000000022413

**Published:** 2020-09-25

**Authors:** Shuliang Chen, Chunye Ma, Ce Zhang, Rui Shi

**Affiliations:** a Department of Neurology; b Clinical Drug Trial Institution, The Second Hospital of Dalian Medical University, Dalian, China.

**Keywords:** early ischemic stroke, non-invasive factors, risk prediction, nomogram, screening

## Abstract

Stroke is the acute onset of neurological deficits and is associated with high morbidity, mortality, and disease burden. In the present study, we aimed to develop a scientific, nomogram for non-invasive predicting risk for early ischemic stroke, in order to improve stroke prevention efforts among high-risk groups. Data were obtained from a total of 2151 patients with early ischemic stroke from October 2017 to September 2018 and from 1527 healthy controls. Risk factors were examined using logistic regression analyses. Nomogram and receiver operating characteristic (ROC) curves were drawn, cutoff values were established. Significant risk factors for early ischemic stroke included age, sex, blood pressure, history of diabetes, history of genetic, history of coronary heart disease, history of smoking. A nomogram predicting ischemic stroke for all patients had an internally validated concordance index of 0.911. The area under the ROC curve for the logistic regression model was 0.782 (95% confidence interval [CI]: 0.766–0.799, *P* < .001), with a cutoff value of 2.5. The nomogram developed in this study can be used as a primary non-invasive prevention tool for early ischemic stroke and is expected to provide data support for the revision of current guidelines.

## 1 Introduction

Stroke refers to the acute onset of neurological deficits due to impairments in local blood circulation, with symptoms lasting at least 24 hours.^[1–3]^ The incidence of ischemic stroke is higher than that of hemorrhagic stroke, accounting for 60% to 70% of all stroke cases.^[2]^ Stroke is associated with high morbidity, mortality, and disease burden, significantly impacting both individual families and society.^[3]^ The disease burden of stroke continues to increase worldwide, further exacerbating the adverse socio-economic effects of the disease.^[4]^ The World Health Organization has predicted that if the mortality rate remains unchanged, nearly 4 million people will die from stroke each year in China by 2030. Thus, stroke prevention is critical for promoting human health and reducing social burden. Non-invasive methods for actively assessing stroke risk are required to ensure appropriate intervention and stroke control in high-risk groups.^[5]^

The 2018 Guidelines for Early Treatment of Acute Ischemic Stroke issued by the American Heart Association/American Stroke Association,^[6]^ and the 2018 Guidelines for the Diagnosis and Treatment of Acute Ischemic Stroke issued by the Chinese Medical Association^[7]^ have highlighted the need for active control of vascular risk factors for effective stroke prevention. Despite a thorough review, the authors of these guidelines failed to identify a scientific, non-invasive scale for assessing early ischemic stroke risk. Given the urgent need for a practical, non-invasive system for evaluating stroke risk, we developed a nomogram based on the characteristics of ischemic stroke and related factors.^[8]^ Self-assessments can be performed at home, providing a basis for the prevention of ischemic stroke in high-risk individuals. In the present study, we used this nomogram to compare data between patients with early ischemic stroke and healthy controls.

## 2 Methods and materials

### 2.1 Date collection

We collected all patients data from 2151 patients with early ischemic stroke treated at our neurology department from October 1, 2017 to September 30, 2018. The patient sample included 1289 men (59.92%) and 862 women (40.08%), with an average age of 66.9 ± 11.9 years. A simple random sampling method was used to collect data from 1527 healthy controls, including 787 men (51.53%) and 740 women. The average age of the controls was 59.6 ± 13.9 years.

### 2.2 Investigation method

We collected data regarding the following input variables: sex (male/female), age (years), height (cm), weight (kg), history of smoking (yes/no), history of alcohol use (yes/no), history of hypertension (yes/no), history of diabetes (years), family history (yes/no), history of coronary heart disease (yes/no), bilateral arterial blood pressure (mmHg), and pulse (beats/min). The occurrence of ischemic stroke was regarded as the output variable. All data were desensitized and encrypted to remove identifying information. Thus, the requirement for informed consent was waived by the hospital ethics committee. Ethics committee approval no. 90, 2017, review by ethics committee of the Second Affiliated Hospital of Dalian Medical University.

### 2.3 Inclusion and exclusion criteria

All included patients were diagnosed with early ischemic stroke in accordance with 2018 American Heart Association/American Stroke Association guidelines.^[6]^ Patients were diagnosed based on acute onset, the presence of focal neurological deficits, imaging lesions, or symptoms/physical signs persisting for >24 hours. Non-vascular causes and cerebral hemorrhage were excluded based on brain computed tomography (CT)/magnetic resonance imaging (MRI) findings. Additional inclusion criteria were as follows: first initial ischemic stroke and complete information regarding the factors investigated.

Patients with incomplete data or data that could not be effectively desensitized were excluded, along with those who had cerebral infarction in combination with other diseases, except for diabetes, high blood pressure, and high blood lipid levels. Patients with a National Institutes of Health Stroke Scale (NIHSS) score ≥16 points were also excluded.

Inclusion criteria for the healthy control group were as follows: no abnormalities during routine health examinations and complete information regarding all factors investigated. Those with incomplete information, information that could not be effectively desensitized, or a history of disease based on hospital medical records were excluded.

In accordance with these inclusion and exclusion criteria, data were extracted from the Hospital Information System using the SQL language.

### 2.4 Statistical analysis

SPSS version 13.0 (Chicago, IL, USA) was used for analyses. The measurement data were expressed as the mean ± standard deviation. Two independent samples *t* tests were used to compare data between the groups. Count data were expressed as quantities, and the relevant comparisons were performed using Chi-square tests. The level of statistical significance was set at *P* < .05. Modeling analyses were performed using the Backward LR method in SPSS Modeler 14.1. To calculate the accuracy of the model, the inclusion and exclusion criteria were set to 0.05 and 0.1, respectively. Receiver operating characteristic (ROC) curves were then drawn, and the cutoff value was calculated.

We used R3.6.1 software to draw the nomogram diagram and calculate the c-index.

## 3 Results

### 3.1 General characteristics

The general characteristics of the patient and control groups are presented in Table 1. Non-invasive factors exhibiting significant differences between the groups were included in the multivariate model (*P* < .05).

**Table 1:**
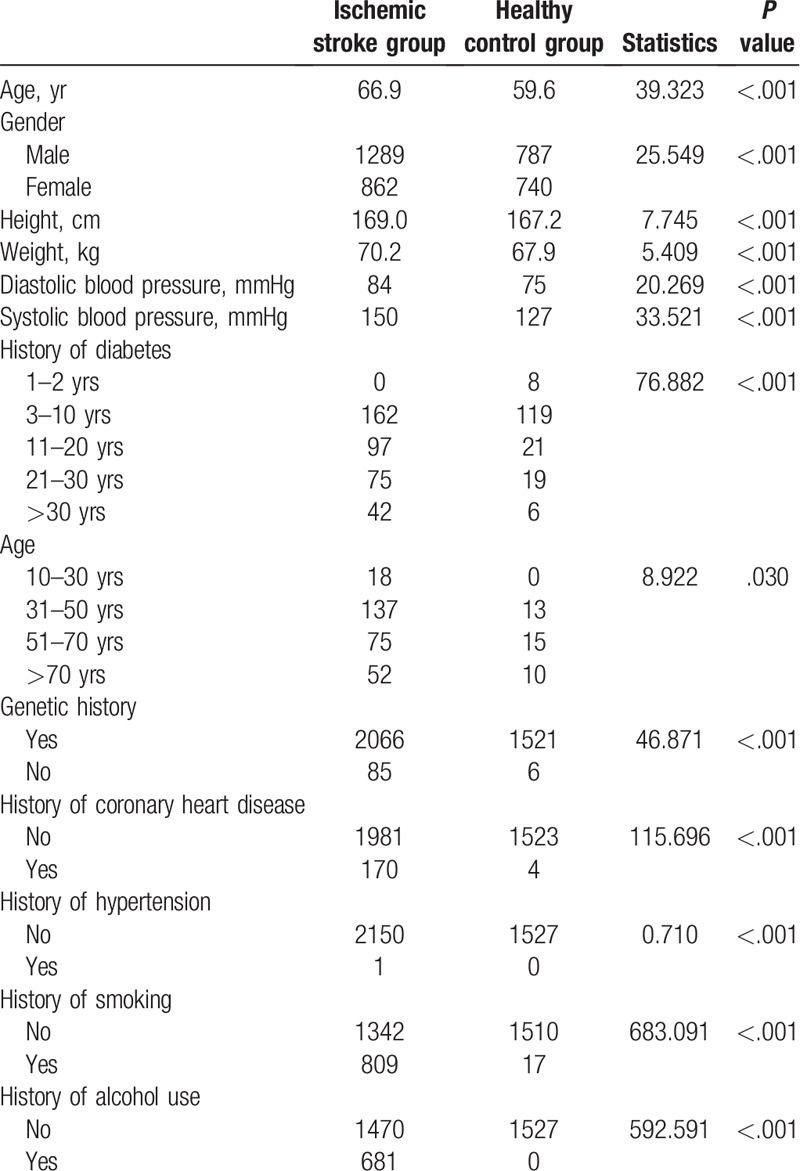
Screening for non-invasive risk factors for early ischemic stroke.

### 3.2 Results of logistic regression analysis

The 13 non-invasive variables identified in Section 3.1 were included in the model for assessing ischemic stroke. A binomial modeling procedure was adopted, and main effects were examined while considering the significance threshold. The significance threshold was set to 0.05, the maximum iteration number was 20, and the maximum stepwise dichotomy was 5. Following Backward LR analysis, the following 6 factors were excluded: sex, blood pressure, genetic history, coronary heart disease history, smoking history, and diabetes history. Age was entered into the final model and was found to be statistically significant. The regression model fits good, and the results of the analysis are shown in Table 2.

**Table 2:**
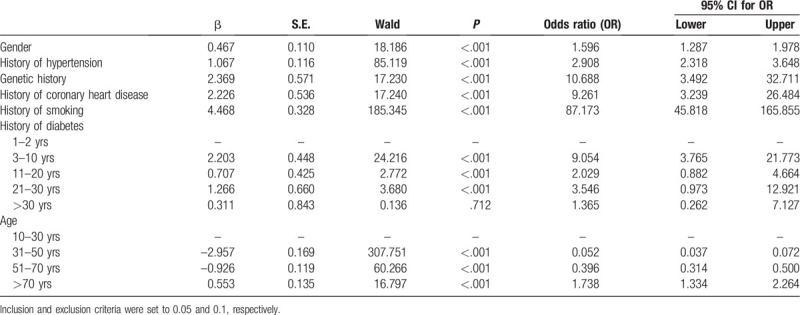
Results of multivariate logistic regression analysis.

### 3.3 Non-invasive nomogram for risk prediction

**Figure 1:**
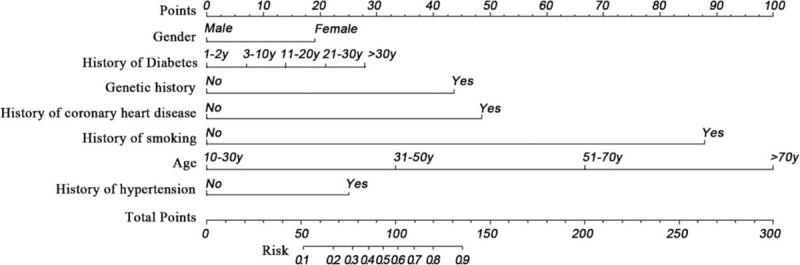
Nomogram non-invasive predicting risk for early ischemic stroke. Non-invasive factor including gender, history of diabetes, genetic history, history of coronary heart disease, history of smoking, age. After the scores of all factors were calculated and the total score was added up, the risk value of ischemic stroke was obtained by the total score corresponding to the risk score.

Based on the results of the logistic regression analysis, a non-invasive nomogram was developed using data for all patients based on 7 standard parameters. The internally validated c-index of this nomogram was 0.911 (Fig. 1). In leave-one-out cross-validation, the c-index for models based on data ranged from 0.869 to 0.967, suggesting similar model performance across different patient groups.

**Figure 2:**
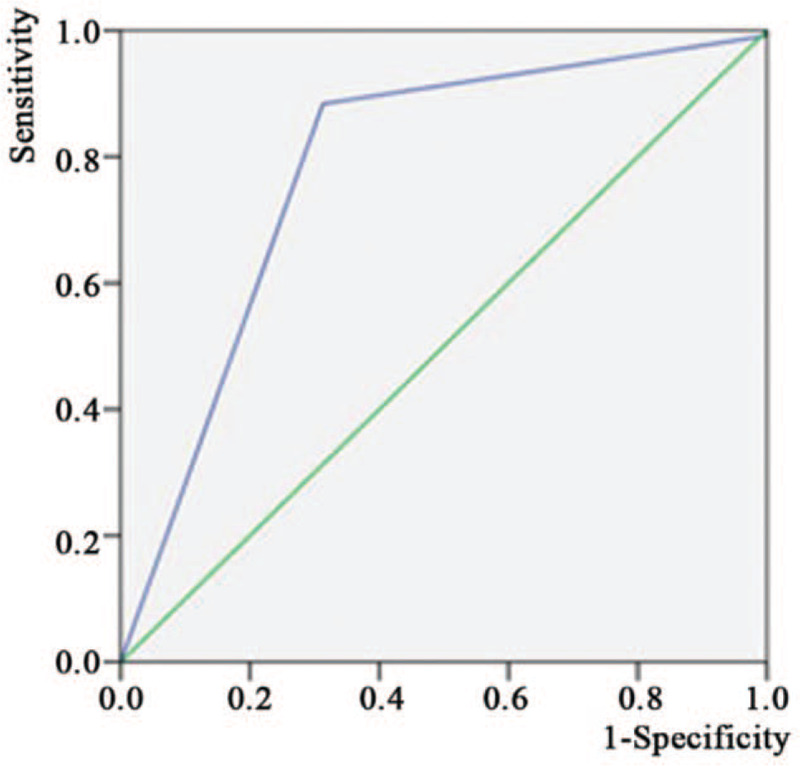
ROC curve of early ischemic stroke predicts model. The area under curve of ROC for early ischemic stroke risk predicted model is 0.782 (95% CI: 0.766–0.799, *P* < .001). CI = confidence interval; ROC = receiver operating characteristic.

The area under curve of ROC for early ischemic stroke risk predicted model is 0.782 (95% confidence interval [CI]: 0.766–0.799, *P* < .001), and the ROC curve is shown in Fig. 2. Based on the largest Youden index, the cutoff value of the final scale was 2.5. High model accuracy was observed in model, ranging from 78.91% to 80.76% (Table 3).

**Table 3:**
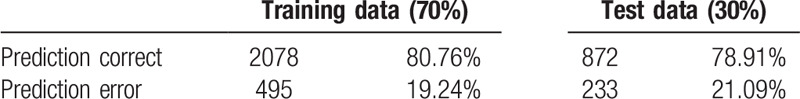
Predictive accuracy (n = 3678).

## 4 Discussion

In the present study, we analyzed real-world clinical data to develop a non-invasive screening tool for early ischemic stroke, which achieved a prediction accuracy of up to 80.76%. Our nomogram could be used to non-invasive screening for early ischemic stroke patients to determine the need for deep therapy. Patient at low risk of early ischemic stroke may be managed expectantly to avoid the potential therapy. By contrast these at high risk of ischemic stroke may be candidates for early therapy. Thus, this nomogram can be used to aid in level 1 prevention of ischemic stroke, and will undoubtedly provide useful information for reducing morbidity and disease burden. In addition to its non-invasive nature and high degree of accuracy, our scale is advantageous in that it is convenient, easy to use, and associated with lower costs than more invasive screening measures. Big data mining may also aid in making monogram more accurate and effective. Furthermore, nomogram can be disseminated to non-professionals via the media or internet within a short period of time, increasing public awareness of risk factors for ischemic stroke.^[9]^

In accordance with previous findings, our study supports the notion that identifying patients at high risk for early ischemic stroke and taking active and effective preventive measures is critical for controlling stroke incidence.^[10]^ Such preventive measures can reduce the incidence of ischemic stroke and decrease morbidity, disability, and mortality rates.^[2]^ For example, modifying lifestyle factors may aid in preventing stroke in high-risk populations. As >76% of strokes are first-episode cases, effective primary prevention is especially important for reducing the incidence of stroke.^[11]^

Our results indicated that advanced age is among the most important factors influencing the development of ischemic stroke. Age ranging from 31 to 50 years or 51 to 70 years was negatively correlated with the occurrence of early ischemic stroke, while a positive correlation was observed in patients over 70 years of age. Indeed, recent studies have indicated that young and middle-aged patients account for only 9.5% to 17.4% of all cases of cerebrovascular disease.^[12]^ However, our analysis indicated that smoking exerted the greatest impact on the onset of ischemic stroke. Although the dangers of smoking are well known and the benefits of smoking cessation have been widely confirmed, smoking is not well controlled among Chinese patients with stroke, especially among patients over 40 years of age: although the smoking age has increased, the average smoking rate has increased as well.^[7]^

In accordance with previous research, our data indicated that hypertension is a key risk factor for early ischemic stroke.^[13–16]^ Effective control of high blood pressure may therefore reduce the risk of early ischemic stroke. We also observed that coronary heart disease significantly increased the risk of ischemic stroke. Given that previous studies^[17]^ have reported that patients with ischemic stroke exhibit coronary artery lesions with obvious clinical symptoms, treatment and prevention of coronary heart disease may reduce ischemic stroke risk as well. In addition, studies have revealed that hyperglycemia leads to lipid condensation in the vascular endothelium and that atheroma plaque cleavage leads to the occurrence of ischemic stroke.^[18]^ Epidemiological studies have further indicated that ischemic stroke tends to occur within families.^[19]^ In accordance with this finding, we observed an increased risk of ischemic stroke in patients with a family history of ischemic stroke. Thus, monitoring and prevention efforts should also focus on relevant family history.

Although the present study included a large sample of patients and controls, further large-scale, multicenter studies are required to improve the accuracy of the nomogram and the robustness/universality of the model. Such studies will provide a more reliable and comprehensive basis for controlling early ischemic stroke.

In this study, we developed a non-invasive screening tool for ischemic stroke using clinical data, the prediction accuracy ranged from 78.91% to 80.76%. Our findings may support the revision of current guidelines for stroke prevention. But the study also had several limitations. First, the study was a single-centered observational design. Second, given the nature of the study, plausible mechanisms for Ischemic Stroke were not addressed. Third, the accuracy of the model needs to be improved. Further studies are needed to improve the prediction effect of the model. Make better improvement on the effect of the nomogram.

## 5 Conclusion

The nomogram developed in this study can be used as a primary non-invasive prevention tool for early ischemic stroke and is expected to provide data support for the revision of current guidelines.

## Acknowledgments

The authors would like to thank Editage [www.editage.cn] for English language editing.

## Author contributions

**Formal analysis:** Ce Zhang.

**Investigation:** Shulaing Chen, Ce Zhang.

**Project administration:** Ce Zhang.

**Software:** Rui Shi.

**Validation:** Chunye Ma, Shuliang Chen.

**Visualization:** Rui Shi, Ce Zhang.
